# Drug use patterns associated with risk of non-adherence to antiretroviral therapy among HIV-positive illicit drug users in a Canadian setting: a longitudinal analysis

**DOI:** 10.1186/s12879-015-0913-0

**Published:** 2015-04-18

**Authors:** Pouya Azar, Evan Wood, Paul Nguyen, Maxo Luma, Julio Montaner, Thomas Kerr, M-J Milloy

**Affiliations:** BC Centre for Excellence in HIV/AIDS, St. Paul’s Hospital, 608-1081 Burrard Street, Vancouver, BC Canada; Division of AIDS, Department of Medicine, University of British Columbia, Vancouver, BC Canada

**Keywords:** HIV, Antiretroviral therapy, Illicit drug use, Heroin, Cocaine, Adherence

## Abstract

**Background:**

Among people living with HIV/AIDS, illicit drug use is a risk for sub-optimal treatment outcomes. However, few studies have examined the relative contributions of different patterns of drug use on adherence to antiretroviral therapy (ART). We sought to estimate the effect of different types of illicit drug use on adherence in a setting of universal free HIV/AIDS treatment and care.

**Methods:**

Using data from ongoing prospective cohorts of HIV-positive illicit drug users linked to comprehensive pharmacy dispensation records in Vancouver, Canada, we examined factors associated with ≥95% prescription refill adherence using generalized estimating equations (GEE) logistic regression.

**Results:**

Between 1996 and 2013, 692 ART-exposed individuals were followed for a median of 42.7 months (Interquartile Range: 29.1–71.7). In multivariable GEE analyses, heroin injection (Adjusted Odds Ratio [AOR] = 0.75, 95% Confidence Interval [CI]: 0.66–0.85) as well as cocaine injection (AOR = 0.80, 95% CI: 0.72–0.90) were associated with lower likelihoods of optimal adherence. Methadone maintenance therapy (AOR = 1.88, 95% CI: 1.68–2.11) was associated with a greater likelihood of adherence.

**Conclusions:**

Periods of heroin and cocaine injection appeared to have the most deleterious impact upon antiretroviral adherence. The findings point to the need for improved access to treatment for heroin use disorder, particularly methadone, and highlight the need to identify strategies to support ART adherence among cocaine injectors.

## Background

The development of antiretroviral therapy (ART) has led to substantial declines in HIV/AIDS-associated morbidity and mortality among many groups in many settings worldwide [1]. Adherence to ART is the primary factor determining the degree and durability of optimal response to treatment, including achieving non-detectable levels of plasma HIV RNA. [2]. Studies indicate that up to 95% adherence is required to achieve maximum viral load suppression [3]. Suboptimal adherence is associated with an increased risk of HIV/AIDS-associated mortality and morbidity [3] and elevated rates of viral resistance to treatment [4].

In many studies, active illicit drug use among individuals living with HIV/AIDS is associated with decreased access to HIV treatment, reduced medication adherence and increased mortality [5]. Although many studies have focused on the impact of heroin use on the clinical management of HIV infection [6,7] fewer studies have examined the influence of distinct illicit drug use patterns. Thus, we sought to examine the relative contribution of different illicit drug use patterns on ART non-adherence among a group of HIV-positive illicit drug users.

## Methods

Data for these analyses were derived from the Vancouver Injection Drug Users Study (VIDUS) and AIDS Care Cohort to evaluate Exposure to Survival Services (ACCESS), two ongoing prospective observational cohorts of illicit drug users in Vancouver, Canada. Beginning in 1996, persons who had injected illicit drugs other than cannabinoids in the previous month were recruited into VIDUS. Since 2005, persons who used illicit drugs and were HIV-seropositive at baseline or during the study are subsequently followed in ACCESS. Both cohorts use identical recruitment and follow-up procedures to allow for combined analyses.

Described in detail previously [8-10], the cohorts were populated through snowball sampling and extensive street outreach in the city’s Downtown Eastside neighbourhood, an area with an open drug market and high levels of injection drug use, poverty and HIV infection [11,12]. Participants of these studies were 18 years of age or older and provided written informed consent. At baseline and every six-month follow-up interview, participants answered a standardized interviewer-administered questionnaire, were examined by a study nurse and provided blood samples for serologic analysis. Participants are actively referred to primary care services and drug treatment programs where available and were provided with a $30 stipend per study visit. A unique feature of these studies is that the Canadian province of British Columbia delivers all HIV care including medications at no charge and employs a system in which every instance of antiretroviral dispensation and all HIV clinical monitoring is captured through a centralized registry [8,13,14]. This allows a comprehensive retrospective and prospective clinical HIV profile for all participants. Ethics approval has been provided annually by University of British Columbia/Providence Health Care Research Ethics Board.

The present analyses considered all HIV-seropositive participants who had been dispensed at least one day of antiretroviral therapy (ART) between May 1996 and May 2013. The primary outcome of interest in this study was adherence to ART in the six month period prior to each study interview. As in previous studies [9,15], we estimated antiretroviral adherence using a validated measure based on pharmacy refill records provided by the confidential record linkage with the database of the Drug Treatment Program. Specifically, adherence was estimated by calculating the number of days for which an individual was dispensed ART in the previous six months over the number of days since they had initiated ART, capped at 180 days. We dichotomized this measure as ≥95% vs. <95%. The cut-off threshold of 95% was chosen as this level has previously been shown to be closely associated with virological suppression and survival [15,16].

Explanatory variables of interest included socio-demographic data: age (per 10 years older), gender (female vs. male), and Caucasian ancestry (yes vs. no). Variables related to drug use characteristics included: heroin injection (yes vs. no), cocaine injection (yes vs. no), amphetamine injection (e.g., “speed”, “uppers”, crystal methamphetamine; yes vs. no), crack cocaine smoking (yes vs. no), and enrollment in methadone maintenance therapy (yes vs. no). All drug use characteristics defined above were treated as time-updated based on questionnaire data pertaining to the six month period prior to each study interview [8,13]. We also included CD4 cell count (per 100 cells/μL increase) at ART initiation using the last CD4 cell count observation conducted prior to the first dispensation of ART. We also included a time-updated variable measuring the time since the participant initiated antiretroviral therapy (per year increase), using records from this setting’s comprehensive antiretroviral dispensary.

As a first step, we visually inspected trends in the proportion of all participants reporting heroin injecting, cocaine injecting, crack smoking and amphetamine injecting at each interview period. Next, we visually inspected changes in median adherence rate at each interview period over time.

Since serial measures for each subject (i.e., multiple 6-month observation periods) were available for many participants, we estimated the relationships between different socio-demographic, drug use and clinical factors and adherence using generalized estimating equations (GEE) with a logit-link function. As a first step, we conducted bivariable GEE analyses to determine which variables were statistically associated with adherence in unadjusted analyses. To adjust for potential confounding and identify factors that were associated with the outcome, all significant variables in the bivariable analyses were considered in the full multivariable model. With the drug use patterns being forced into the model, a backwards model selection procedure was used to identify the multivariate model with the best overall fit as indicated by the lowest quasilikelihood under the independence model criterion value. All statistical analyses were performed using the SAS software version 9.3 (SAS, Cary, NC). All *p*-values are two-sided.

## Results

**Figure 1:**
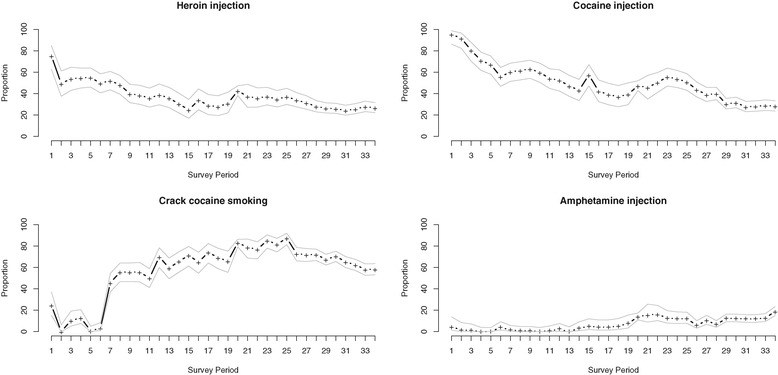
Drug use patterns over time; Proportion of all participants reporting heroin injection, cocaine injection, crack cocaine smoking and amphetamine injection in the previous 180 days at each survey period (plus symbol), with 95% confidence intervals.

**Figure 2:**
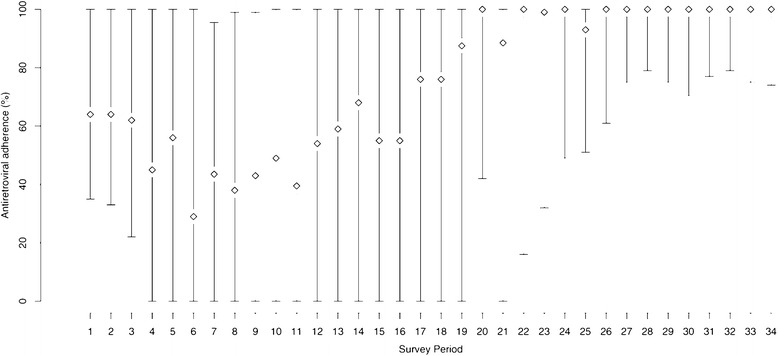
Antiretroviral adherence patterns over time; Median antiretroviral adherence rate achieved in the last 180 days among all participants at each survey period (diamond), with lower and upper quartiles.

During the study period, 692 individuals were included in these analysis, among whom 213 (30.8%) were female, the median age at baseline was 42 years (Inter-quartile Range (IQR): 35–47) and the median follow-up duration was 42.7 months (IQR: 29.1–71.7). At baseline (Table 1), 338 (48.8%) individuals reported heroin injection in the previous six months, 388 (56.1%) reported cocaine injection in the previous six months, 84 (12.1%) reported amphetamine injection in the previous six months and 451 (65.2%) reported smoking crack cocaine in the previous six months. The proportions of participants at each interview period reporting these drug use patterns are shown in Figure 1. Figure 2 depicts the median adherence rate for all participants at each interview period as well as the lower and upper quartiles. Over the entire study period, the median adherence rate was 98% (Inter-Quartile Range [IQR]: 37, 100). Among all observation periods, 3073 (51%) were characterized by ≥95% adherence. Correlation between the adherence rate and plasma HIV-1 RNA viral load (log10 transformed) was −0.67; correlation between the adherence rate and CD4+ cell count was 0.17.

**Table 1:** **Baseline characteristics of 692 HIV-positive illicit drug users stratified by adherence to ART in the previous six months**

**Characteristic**	**<95% adherence**	**≥95% adherence**	**OR** ^**1**^	**95% CI** ^**2**^	***p*** **value**
	**301 (43.5) n (%)**	**392 (56.6) n (%)**			
Age					
Median (IQR)	40 (34 – 45)	43 (36 – 48)	1.05	1.02 – 1.06	<0.001
Gender					
Male	195 (64.8)	284 (72.6)	1.00		
Female	106 (35.2)	107 (27.5)	0.69	0.50 – 0.96	0.027
Caucasian ancestry					
No	143 (47.5)	140 (35.8)	1.00		
Yes	158 (52.5)	251 (64.2)	1.62	1.19 – 2.20	0.002
Heroin injection^3^					
No	141 (47.0)	211 (54.1)	1.00		
Yes	159 (53.0)	179 (45.9)	0.75	0.56 – 1.02	0.064
Cocaine injection^3^					
No	126 (41.9)	178 (45.5)	1.00		
Yes	175 (58.1)	213 (54.5)	0.86	0.64 – 1.17	0.336
Amphetamine injection^3^					
No	271 (90.0)	337 (86.2)	1.00		
Yes	30 (10.0)	54 (13.8)	1.45	0.90 – 2.33	0.125
Crack cocaine smoking^3^					
No	107 (35.5)	134 (34.2)	1.00		
Yes	194 (64.5)	257 (65.7)	1.06	0.77 – 1.45	0.727
MMT^3^					
No	202 (67.1)	210 (53.7)	1.00		
Yes	99 (32.9)	181 (46.3)	1.76	1.29 – 2.40	<0.001
Time since ART initiation (per year)	5.1 (2.5 – 8.9)	6.5 (3.1 – 11.4)	0.94	0.91 – 0.97	<0.001
Median (IQR)					
CD4+ cells at ART initiation					
Median (IQR)	260 (150 – 390)	220 (140 – 360)	1.00	1.00 – 1.00	0.074

1. Odds Ratio; 2. 95% Confidence Interval; 3. Refers to 180 day period prior to the baseline interview.

The bivariable and multivariable GEE analyses of adherence to ART are shown in Table 2. In the final multivariable model, heroin injection (Adjusted Odds Ratio [AOR] = 0.76, 95% Confidence Interval [CI]: 0.67–0.85), cocaine injection (AOR = 0.74, 95% CI: 0.66–0.83), female sex (AOR = 0.77, 95% CI: 0.68 – 0.87) and CD4 cell count at ART initiation (AOR = 0.87, 95% CI: 0.83 – 0.92) were negatively associated with optimal adherence to ART. Older age (AOR = 1.66, 95% CI: 1.54 – 1.80) and use of methadone maintenance therapy (AOR = 1.88, 95% CI: 1.68 –2.11) were independently and positively associated with optimal adherence to ART.

**Table 2:** **Univariable and multivariable generalized estimating equations analyses of factors associated with ≥ 95% adherence to antiretroviral therapy among 692 illicit drug users**

**Characteristic**	**OR** ^**1**^	**95% CI** ^**2**^	**p**	**AOR** ^**3**^	**95% CI** ^**2**^	**p**
Gender (female vs. male)	0.70	0.56 – 0.88	0.002	0.77	0.68 – 0.87	0.038
Age (per 10 years older)	2.04	1.75 – 2.38	<0.001	1.66	1.54 – 1.80	<0.001
Caucasian (yes vs. no)	1.37	1.12 – 1.69	0.002	1.07	0.95 – 1.19	0.571
Heroin injection (yes vs. no)^4^	0.67	0.58 – 0.78	<0.001	0.76	0.67 – 0.85	0.002
Cocaine injection (yes vs. no)^4^	0.73	0.63 – 0.85	<0.001	0.74	0.66 – 0.83	0.001
Amphetamine injection (yes vs. no)^4^	1.14	0.89 – 1.47	0.305			
Crack cocaine smoking (yes vs. no)^4^	0.95	0.82 – 1.10	0.525			
Methadone maintenance therapy (yes vs. no)^4^	1.74	1.45 – 2.10	<0.001	1.96	1.75 – 2.19	<0.001
Time since ART initiation (per year increase)	1.08	1.06 – 1.11	<0.001	1.02	0.99 – 1.04	0.090
CD4+ cell count at ART initiation (per 100 cells/mL increase)	0.87	0.83 – 0.92	<0.001	0.88	0.85 – 0.90	<0.001

1. Odds Ratio; 2. 95% confidence interval; 3. Adjusted Odds Ratio; 4. Time-updated, refers to the six-month period prior to the interview.

## Discussion

In this long-running community-recruited study of illicit drug users linked to comprehensive HIV clinical records in a setting of universal free HIV/AIDS treatment and care, we observed that periods of heroin and cocaine injection were independently and negatively associated with the likelihood of optimal adherence to ART. We did not observe a statistical relationship between periods of either amphetamine injection or crack cocaine smoking and the likelihood of optimal adherence to ART. Meanwhile, engagement in methadone maintenance therapy was independently and positively associated with optimal adherence to ART.

Our finding of strong and independent links between heroin use, engagement in methadone maintenance and the likelihood of optimal ART adherence suggests that the expansion of evidence-based addiction treatment strategies, such as methadone, will likely improve retention in HIV/AIDS care among opioid-dependent illicit drug users [17-19]. Improving access to methadone will require scale-up treatment slots in settings where methadone is currently available and removal of legal impediments to this evidence-based opioid treatment modality [20].

In our study, we did not observe a statistical relationship between amphetamine use and adherence to ART. Crystal methamphetamine use is particularly prevalent among some populations of people living with HIV/AIDS and persons at risk for HIV, especially men who have sex with men. [21-23] Numerous studies have found that, among this population, amphetamine use in is linked to decreased medication adherence as a result of binging episodes, and in the long-term, has been associated with the development of antiretroviral-resistant viral strains [23]. In the current study, which contained a high proportion of individuals reporting periods of poly-substance use, this association was not observed. This might reflect the relatively smaller number of individuals reporting periods of amphetamine injection and with cohorts of men who have sex with men, which would be more heterogeneous with respect to drug use. Instead, the present study observed a strong and independent link between injection cocaine use and sub-optimal adherence, underling the urgent need for effective pharmacotherapies to address stimulant use, especially in the context of HIV disease.

Although we observed increasing levels of adherence over time, a substantial proportion of all participants displayed sub-optimal adherence to prescribed treatment at every interview period. Our model indicates that specific drug use patterns each had different relationships to antiretroviral adherence. For example, while periods of heroin injection were 23% less likely to be characterized by ≥95% adherence, there was no significant difference between levels of optimal adherence during periods of amphetamine injection. Unfortunately, our model does not offer specific insights into possible explanations for these divergent results. Thus, future research should investigate the possible behavioural-, psychologic-, social- and structural-level barriers and facilitators of adherence for different groups of illicit drug users in order to optimize substance abuse treatment and support ART adherence.

There are some limitations to this study to note. Participant selection for this observational study was not random and the results of this study cannot be generalized to the wider population of HIV-positive illicit drug users. Regarding associations drawn from the results of the study, there is potential for unmeasured confounding. In an attempt to minimize the impact of confounding on the observed relationships we used multivariable modelling. Also, we have previously observed that at least 95% adherence to prescribed ART is strongly associated with viral suppression and survival [15,16]. Newer formulations of ART are more potent and evidence suggests they may deliver comparable rates of viral suppression at lower adherence thresholds [24,25]. Thus, future research might consider the effects of illicit drug use on attaining different adherence thresholds and viral suppression. Finally, we recognize that we are unable to determine the possible temporal relationships between illicit drug use behaviours and antiretroviral dispensation patterns within any 180-day observation period.

## Conclusions

To conclude, our study utilized data from a long-running community-recruited prospective cohort of HIV-seropositive illicit drug users in the setting of free and universal access to HIV care. Periods of injection heroin use and injection cocaine use were both independently and negatively associated with a lower likelihood of optimal adherence to ART while engagement in MMT was associated with higher levels of optimal adherence. Given the importance of addressing substance use in the community and the proven success of MMT at reducing the risk of drug-related harms, our findings support the need to spur efforts to improve access to treatment for problematic substance use among individuals living with HIV/AIDS.
